# Editorial: For a sustainable future: novel insights into agronomically important traits in cereal crops

**DOI:** 10.3389/fpls.2023.1197864

**Published:** 2023-04-18

**Authors:** Quan Xu, Yuanhu Xuan, Hiroki Saito, Shan Li

**Affiliations:** ^1^ Rice Research Institute of Shenyang Agricultural University, Shenyang, China; ^2^ Plant Protection College of Shenyang Agricultural University, Shenyang, China; ^3^ Tropical Agriculture Research Front (TARF), Japan International Research Center for Agricultural Sciences (JIRCAS), Ishigaki, Japan; ^4^ State Key Laboratory of Crop Genetics & Germplasm Enhancement and Utilization, Nanjing Agricultural University, Nanjing, China

**Keywords:** crops, yield, quality, lodging, biotic and abiotic stresses, intercropping, zinc finger proteins (ZFPs)

Since the global population is expected to reach 9.7 billion by 2050, increased cereal crop yield will have a significant positive impact on global food security. However, crop production is threatened by climate change, depletion of fertilizer feedstock, and a number of biotic and abiotic stresses. To achieve sustainable agricultural performance while overcoming these challenging situations, it is critical to understand the molecular mechanisms of agronomically important traits in cereal crops. Recently developed techniques in molecular biology, including genomics and other omics, can reveal the complex molecular mechanisms involved in the control of agronomic traits, providing novel insights into the yield, resistance to biotic and abiotic stresses, and responses to variable environments in cereal crop production.

Rice is one of mankind’s major food staples. Given continuing population growth and increasing competition for arable land between food and energy crops, food security is becoming an ever more serious global problem. Palanog et al. carried out Multi-Cross QTL analysis and Inclusive Composite Interval Mapping for 11 agronomic traits using four connected RILs populations of rice, and detected MC-156 QTLs for agronomic (115) and biofortification (41) traits. Grain weight is a major determinant in rice yield, which is tightly associated with grain size. The major effect QTL identified for biofortification and agronomic traits can be utilized in breeding. Ye et al. reported a nine-base pair deletion in a kinesin-like protein *BRITTLE CULM12* encoding gene (*LGW*) caused a reduced grain length. The overexpression of *LGW* increased the grain length, revealing that *LGW* plays an important role in regulating grain size, and the manipulation of this gene provides a new strategy for regulating grain weight in rice. Grain chalkiness in rice is a highly undesirable trait for human food that negatively affects milling, cooking, eating, and grain appearance. Yang et al. identified two major QTLs for chalkiness, *qPGC5* and *qPGC6* from two single-segment substitution lines (SSSLs) with a significantly lower percentage of grain chalkiness than the recipient parent line. The fine-mapping of the two QTLs will facilitate the cloning of genes for chalkiness and provide new genetic resources to develop new cultivars with low chalkiness even under high-temperature conditions. Huang et al. focused on a superfamily of transcription factors, zinc finger proteins, reviewed the current understanding of zinc finger proteins underlying regulatory network in the regulation of agronomic traits in rice, summarized the current advances in zinc finger proteins, with emphasis on C2H2 and CCCH proteins, and finally discussed their potential in improving rice yield.

Lodging also greatly harmed the crop yield and quality. Niu et al. detected fifteen QTL for stem strength-related traits in wheat using a doubled haploid population derived from a cross between Baiqimai and Neixiang 5, which will be useful for molecular marker-assisted selection for high stem strength and high yield potential. Zhao et al. showed the lodging rate of rice was substantially increased by 81.1% after 11 years of continuous Soil testing formula fertilization using organic fertilizer (STFFOF) treatment. The STFFOF greatly decreases the concentration of Ca, SiO_2_, K, Mg, and non-structural carbohydrates in basal internodes, dramatically increases that of N, P, and weight per ear, but slightly affects the structural carbohydrates.

Crops will face several future challenges including biotic and abiotic stresses that will seriously jeopardize their annual production. Climate change is already threatening agricultural harvest, thereby increasing the risk of food insecurity. Zhang et al. examined the rice photosynthetic parameters, water use efficiency, and yield formation in responses to the co-elevation of [CO_2_] and temperature, provided evidence that the rice genotypic difference in photosynthetic potential under [CO_2_] and temperature co-elevation. Wang et al. provided a full perspective on how drought affects the yield formation of foxtail millet by constructing one work model thereby providing the theoretical foundation for hub genes exploration and drought resistance breeding of foxtail millet. Liu et al. conducted cytological observation and transcriptome analysis to reveal dynamic changes of *Rhizoctonia solani* colonization on leaf sheath and different genes recruited between the resistant and susceptible genotypes in rice, demonstrated that WE-CLSM is a powerful technique for uncovering the mechanism of *R. solani* invading rice and for detecting rice sheath blight–resistant germplasm.

Intercropping is commonly implemented as a way of promoting sustainable agriculture. Dong et al. investigated the process of microbial community assembly in maize, peanuts, and shared rhizosphere soil as well as their regulatory mechanisms on root exudates under different planting patterns by combining metabolomic and metagenomic analyses. The results showed that the yield of intercropped maize increased significantly, while the yield of intercropped peanut significantly decreased. These results indicate that interspecific root interactions improved the soil microenvironment, regulated the absorption and utilization of nitrogen nutrients, and provided a theoretical basis for high yield and sustainable development in the intercropping of maize and peanut.

Elucidation of the molecular basis related to important agronomic traits and improvement of cultivation techniques can help us to meet the challenge of food security. The excellent studies mentioned here demonstrated the importance of the research community in understanding and explaining the novel insights into agronomically important traits in cereal crops for a sustainable future. In the future, technologies such as genotyping, marker-assisted selection, high-throughput phenotyping, genome editing, genomic selection, and *de novo* domestication could enable plant breeders to keep pace with a globally changing environment and increasing population.

## Author contributions

QX: Wrote the draft. YX, HS, and SL: Reviewed and edited the article. All authors contributed to the article and approved the submitted version.

